# A protease activity-based machine-learning approach as a complementary tool for conventional diagnosis of diarrhea-predominant irritable bowel syndrome

**DOI:** 10.3389/fmicb.2023.1179534

**Published:** 2023-07-07

**Authors:** Kazuki Tanaka, Naoki Tanigawa, Isaiah Song, Toru Komatsu, Yugo Kuriki, Yukari Tanaka, Shin Fukudo, Yasuteru Urano, Shinji Fukuda

**Affiliations:** ^1^Institute for Advanced Biosciences, Keio University, Tsuruoka, Yamagata, Japan; ^2^Systems Biology Program, Graduate School of Media and Governance, Keio University, Fujisawa, Kanagawa, Japan; ^3^Transborder Medical Research Center, University of Tsukuba, Tsukuba, Ibaraki, Japan; ^4^Gut Environmental Design Group, Kanagawa Institute of Industrial Science and Technology, Kawasaki, Kanagawa, Japan; ^5^Graduate School of Pharmaceutical Sciences, The University of Tokyo, Hongo, Tokyo, Japan; ^6^Department of Gastroenterology, Sendai Kousei Hospital, Sendai, Miyagi, Japan; ^7^Department of Behavioral Medicine, Tohoku University Graduate School of Medicine, Sendai, Japan; ^8^Graduate School of Medicine, The University of Tokyo, Hongo, Tokyo, Japan; ^9^Laboratory for Regenerative Microbiology, Juntendo University Graduate School of Medicine, Hongo, Tokyo, Japan

**Keywords:** irritable bowel syndrome, trypsin, fluorescent probe, machine-learning, prediction

## Abstract

Irritable bowel syndrome (IBS) has no clinically accepted biomarkers even though it affects a large number of individuals worldwide. To address this lack of understanding, we evaluated peptidase activity in fecal samples from 35 patients with diarrheal IBS without symptom exacerbation (IBS-n) and 35 healthy subjects using a library of 384 fluorescent enzymatic substrate probes. IBS-n patients had high trypsin-like peptidase activity for cleavage of C-terminal lysine and arginine residues and low elastase-like activity for cleavage of C-terminal serine and glycine residues. These fluorescent probe library data, together with diagnostic machine-learning techniques, were able to accurately predict IBS-n. This approach can be used to diagnose diseases where no clinically accepted biomarkers exist, in which fecal enzyme activity is altered and also suggests that the development of new therapies targeting enzyme activities is possible.

## 1. Introduction

Irritable bowel syndrome (IBS) is diagnosed by recurrent abdominal pain or discomfort for at least 3 days per month in the last 3 months, associated with a change in stool frequency or appearance, and no organic cause found. Although IBS affects 3.8–9.2% of the world’s population (Sperber et al., 2017), there are no clinically accepted biomarkers for IBS, and diagnosis is based on self-reported symptoms through a questionnaire such as Rome IV (Menees et al., 2015; Ford et al., 2017; Hou et al., 2021). IBS is thought to have a heterogeneous etiology, and it is not easy to definitively diagnose it as IBS among various diseases with diarrhea symptoms (Longstreth et al., 2006; Enck et al., 2016). Therefore, invasive and burdensome endoscopies are often used to distinguish between IBS and other diseases. As a result, it is necessary to establish a simple diagnostic method for IBS.

Diseases other than IBS also have no clinically accepted biomarkers and require other means of diagnosis. For example, esophageal squamous cell carcinoma can only be diagnosed by invasive endoscopy or biopsy (Cui et al., 2020). However, the enzymatic activity of the serine protease dipeptidylpeptidase IV was recently reported to be elevated in this disease (Onoyama et al., 2016). Indeed, the use of enzymatic reactions as diagnostic tools for certain diseases is an emerging research field (Soleimany and Bhatia, 2020) and may be a solution for diagnosing diseases with otherwise no known biomarkers. In fact, abnormalities in enzymatic reactions have also been reported in IBS patients. For example, trypsin-like protease production from the intestinal epithelium is elevated in IBS patients, and this stimulates intestinal neurons to induce visceral hypersensitivity (Rolland-Fourcade et al., 2017). These findings provide a theoretical basis for treating IBS, and the development of trypsin-targeted drugs is ongoing (Vergnolle, 2016; Rolland-Fourcade et al., 2017). Thus, detecting differences in enzymatic activity may be a possible method of IBS diagnosis as well. Unfortunately, IBS cannot yet be definitively diagnosed on the basis of these enzymatic abnormalities. For greater accuracy, it is necessary to comprehensively measure disease-associated disruptions in enzyme activities using a data-driven approach. However, thousands of enzymes are expressed in human cells, and the functions of many enzymes remain undetermined. In recent years, the alphafold2 platform, which utilizes machine learning, has made it possible to accurately predict protein structures (Jumper et al., 2021). Yet despite such advances, protein and enzyme functions are regulated by multiple factors such as post-translational modifications, protein–protein interactions, and endogenous inhibitors, so protein function cannot be predicted or quantified as easily as that of DNA or RNA (Saghatelian and Cravatt, 2005). A robust method of comprehensively measuring enzyme activity is therefore necessary for development of this potential IBS diagnostic method.

Fluorescent probes based on 7-amino-4-methylcoumarin (Azaryan and Hook, 1994; Drag et al., 2010), Rhodamine Green (Hug et al., 1999) and aminocoumarin (Backes et al., 2000; Tholen et al., 2020) have been developed as methods for quantifying enzyme activity. In this study, we used an HMRG-based probe library with high activation rate and bright fluorescence in the visible light range in a single-step enzymatic reaction (Kuriki et al., 2022). Specifically, a total of 384 HMRG-based fluorescent probe libraries were used to comprehensively measure enzymatic activity in fecal samples from 35 patients with diarrheal IBS (IBS-D) without symptom exacerbation (IBS-n) and 35 healthy subjects. Machine learning was applied to identify enzymatic reactions characteristic of the remission phase of IBS. This diagnostic machine-learning approach could predict IBS with high accuracy.

## 2. Materials and methods

### 2.1. Study subjects and fecal sample collection

A total of 43 male patients with IBS-D [age: mean ± standard deviation (SD), 21.8 ± 1.7 years] who met the Rome III criteria and 40 healthy male controls (age: 22.1 ± 1.3 years) were previously recruited for another study from Tohoku University Hospital between February 2016 and January 2021 (Tanaka et al., 2022). We used fecal samples from 35 healthy individuals and 35 IBS-D patients, from whom we were able to collect a sufficient amount of fecal samples. At the time of study entry, a gastroenterologist skilled in the treatment of IBS interviewed the participants about their symptoms. Subjects with BMI > 25 or < 18.5 kg/m^2^ and those with hyperlipidemia were excluded from the study. Healthy volunteers were subjects without symptoms of functional bowel disorders. None of the IBS-D patients or healthy volunteers had organic diseases or mental disorders. The use of the following drugs was prohibited at least for the indicated week(s) before the experiments; laxatives, antidiarrheals, or probiotics for 1 week and antibiotics, anti-inflammatory drugs, corticosteroids, proton pump inhibitors, transit modulators or tranquilizers for 6 weeks. All participants provided written informed consent for participation in the study, which was approved by the Ethics Committee of the Tohoku University Hospital, Japan (2020-1-578). The IBS Severity Index (IBS-SI) (Shinozaki et al., 2006) was used to assess the participants on the day before the experiment. The patients were asked to record their numerical rating scale of abdominal pain, abdominal discomfort, abdominal bloating, incomplete evacuation, difficulty in passing stool, or dissatisfaction on bowel movements evaluated from 1 (minimum) to 7 (maximum), Bristol Stool Form Scale from 1 (lumpy) to 7 (watery), and number of bowel movements per day for 14 days from the beginning of the study. Fecal samples were collected from healthy volunteers and IBS patients on days when they were experiencing neither abdominal pain or discomfort, nor reporting loose or watery stools. Fecal samples were immediately stored at 4°C, frozen at −80°C within 12 h of collection, and stored at −80°C until sample processing.

### 2.2. Metabolome analysis

Capillary electrophoresis time-of-flight mass spectrometry (CE-TOFMS)-based metabolome analysis of fecal samples was conducted as described previously (Hirayama et al., 2012) with some modifications. In brief, fecal samples were lyophilized using a VD-800R lyophilizer (TAITEC) for 24 h. Freeze-dried feces were disrupted with 3.0-mm Zirconia Beads (Biomedical Science) by vigorous shaking (1,500 rpm for 10 min) using the Shake Master (Biomedical Science). Fecal metabolites were extracted using the methanol:chloroform:water extraction protocol (Wang et al., 2015). CE-TOFMS experiments were performed using the Agilent CE System, the Agilent G3250AA LC/MSD TOF System, the Agilent 1100 Series Binary HPLC Pump, the G1603A Agilent CE-MS adapter, and the G1607A Agilent CE-ESI-MS Sprayer kit. In-house software (MasterHands) (Sugimoto et al., 2010) was used for data processing, quantification, and peak annotation.

### 2.3. Principal coordinates analysis

DNA extraction from fecal samples and sequencing of the 16S rRNA genes were conducted in a previous study (Tanaka et al., 2022). Principal coordinates analysis was performed on fecal microbiome data and metabolome data from healthy subjects (*n* = 35) and IBS-n patients (*n* = 35) using Bray–Curtis dissimilarity.

### 2.4. Protease activity measurement

To evaluate global enzyme activity in fecal samples, freeze-dried samples were suspended with PBS (1 mg feces per mL PBS). Enzyme activity was measured with a plate reader (infinite M200, TECAN) in 384-well black plates with 5 μL/well suspension and 15 μL/well fluorescence probe solution (1.33 μM in PBS containing dimethylsulfoxide as a co-solvent, final, 1 μM). Fluorescence intensity was measured at 30, 60, and 120 min (excitation, 485 nm; emission, 535 nm).

### 2.5. Machine learning-based classification of IBS patients

We implemented the random forest (RF) algorithm with some modifications, based on previous studies (Fukui et al., 2020). RF model was constructed and validated using 384 fluorescence intensity measurements for samples from 70 subjects by splitting training and test samples by a ratio of 7:3. With a training dataset, the number of variables used in each split of a tree was tuned by grid search through five-fold cross-validation. With a test dataset, the final model was used to evaluate performance [accuracy, sensitivity, and Receiver operating characteristic (ROC)]. To assess the robustness of this model’s prediction capability, ROC analysis was applied.

A unified framework of model training and validation and ROC analysis was applied using the R package scikit-learn.

### 2.6. Statistical analysis

Differences between two groups were evaluated with Student’s *t*-test, and differences between groups of three or more were evaluated with Dunnett’s test. Bray–Curtis dissimilarity was evaluated by adonis (also called PERMANOVA) in R package vegan. All statements indicating significant differences reflect a *p* value <0.05.

## 3. Results

### 3.1. Fecal metabolome profiles of IBS patients during the remission phase do not differ from those of healthy subjects

**Figure 1:**
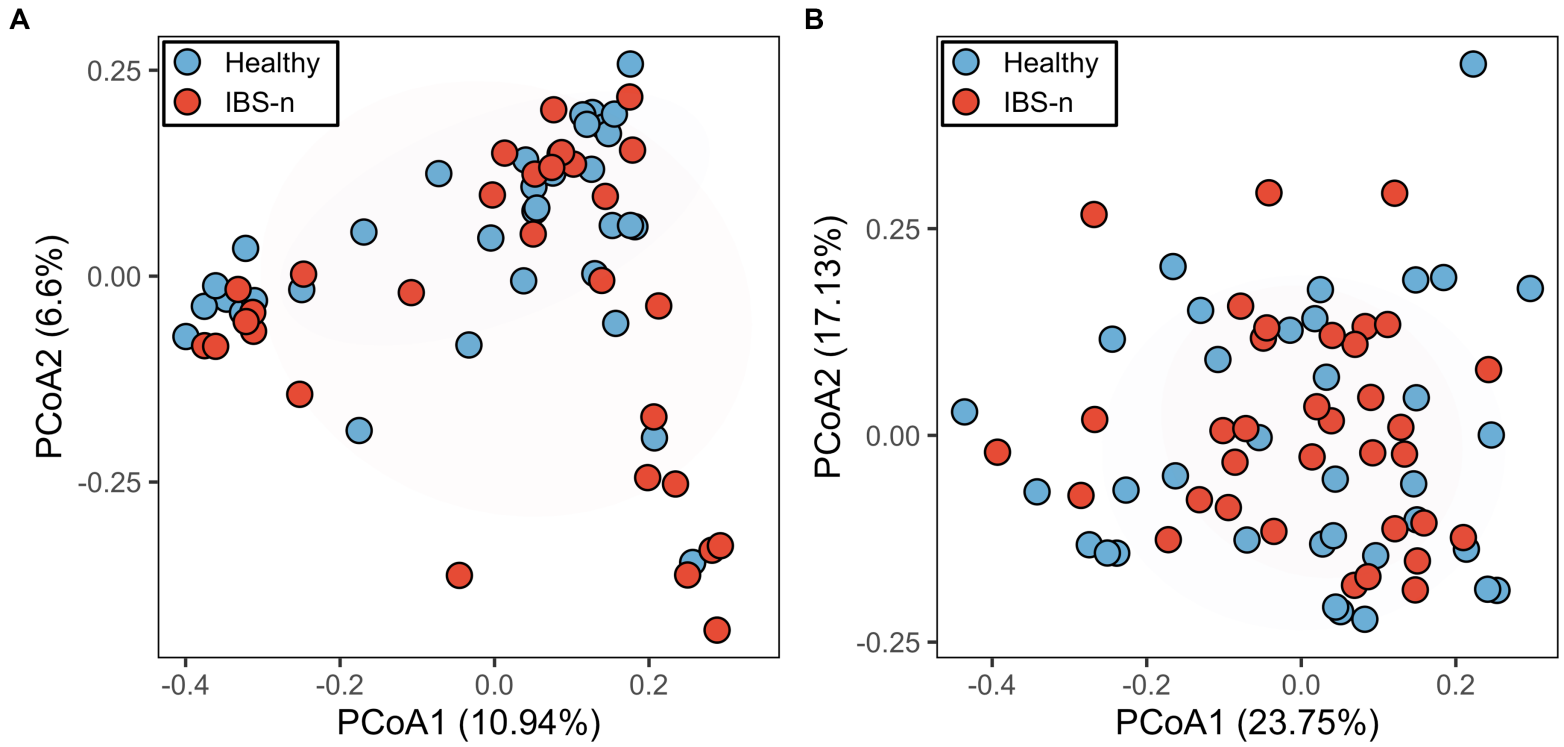
Fecal microbiome and metabolome profiles of IBS-n patients and healthy subjects. Principal coordinates analysis of fecal microbiome data (**A**, *p* = 0.16) and fecal metabolome data (**B**, *p* = 0.45) from healthy subjects (*n* = 35) and IBS-n patients (*n* = 35) using Bray–Curtis dissimilarity. The numbers in parentheses on the axes indicate the contribution ratios.

IBS is characterized by periods of exacerbated symptoms and remission, which makes it difficult to identify a biomarker(s) among cross-sectional studies (Mars et al., 2020). When analyzing only samples from the exacerbation period, it is difficult to identify biomarkers that are specific to IBS-D because diarrhea can have a strong influence, making it difficult to distinguish from other diseases with similar diarrhea symptoms. In contrast, when using samples only from the remission period, it may be difficult to distinguish between remissive and healthy individuals, but this condition can be useful for exploring potential biomarkers. Thus, we used fecal samples collected from a given patient on a day when the patient was experiencing neither abdominal pain nor abdominal discomfort and had not reported loose or watery stools (IBS-n). Although altered gut microbiota composition was thought to be a potential marker for IBS-D, gut microbiota profiles of IBS-n patients did not differ substantially from those of healthy subjects (Figure 1A, *p* = 0.16). To find other candidate markers, we performed a comprehensive metabolomic analysis of feces using CE-TOFMS. A multivariate analysis by principal coordinates analysis revealed little difference between the metabolome profiles of healthy subjects and IBS-n patients (Figure 1B, *p* = 0.45).

### 3.2. Optimal reaction time between the probe library and feces

Due to the observation that the fecal metabolome profiles and fecal microbiome profiles did not differ significantly between healthy subjects and IBS-n patients, we measured the comprehensive enzyme reaction activity in the feces. We used a fluorescent probe of hydroxymethyl rhodamine green, which is quenched by spirocyclic caging but is activated rapidly when an attached dipeptide is hydrolyzed by a peptidase or protease (Urano et al., 2011; Yamashita et al., 2013; Onoyama et al., 2016; Kuriki et al., 2022). The library was comprised of probes possessing dipeptides consisting of various natural and non-natural amino acids to target the reactivities of various peptidases/proteases (Supplementary Figure S1). The rate of each enzymatic reaction—as detected by the cleavage of the 384 probes—depended on the concentrations of the substrates and probes and the enzymatic activity. First, to determine the appropriate reaction time (i.e., after which the probes were mixed with fecal samples), we measured the fluorescence intensity kinetics in a time series using stool samples from 20 individuals (Supplementary Figure S2). We tested reaction times of 0.5, 1, 2, and 3 h and found that 0.5 h was sufficient for detection while being as short as possible.

### 3.3. The enzymomic profile is not significantly altered in IBS-n patients

**Figure 2:**
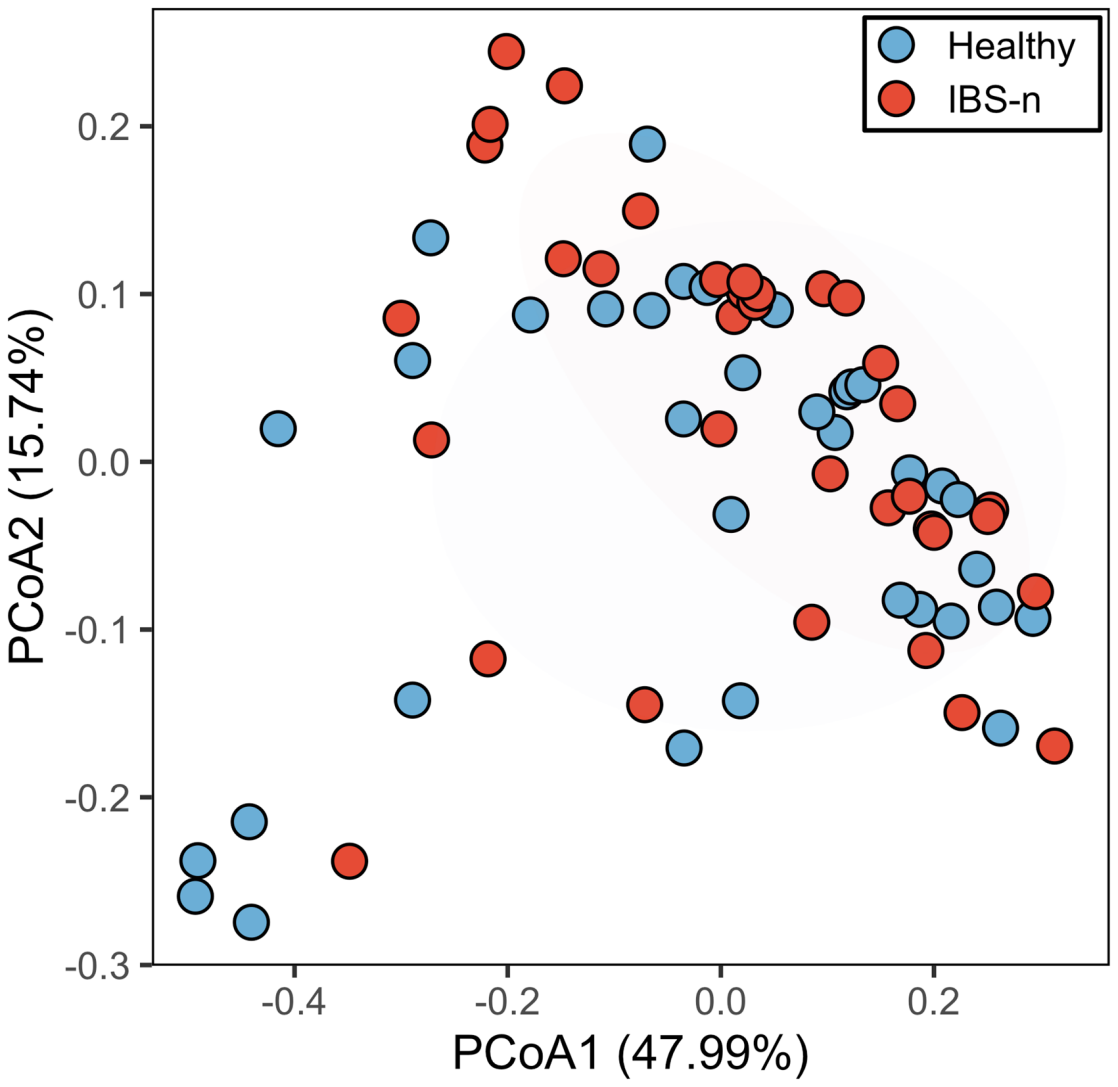
PCoA analysis of probe fluorescence in healthy and IBS-n fecal samples. Analysis using fluorescence intensity data after 0.5 h of mixing 384 probes with feces samples collected from 35 healthy subjects and 35 IBS-n patients. The numbers in parentheses on the axes indicate the contribution ratios. *p* = 0.27, PERMANOVA.

**Figure 3:**
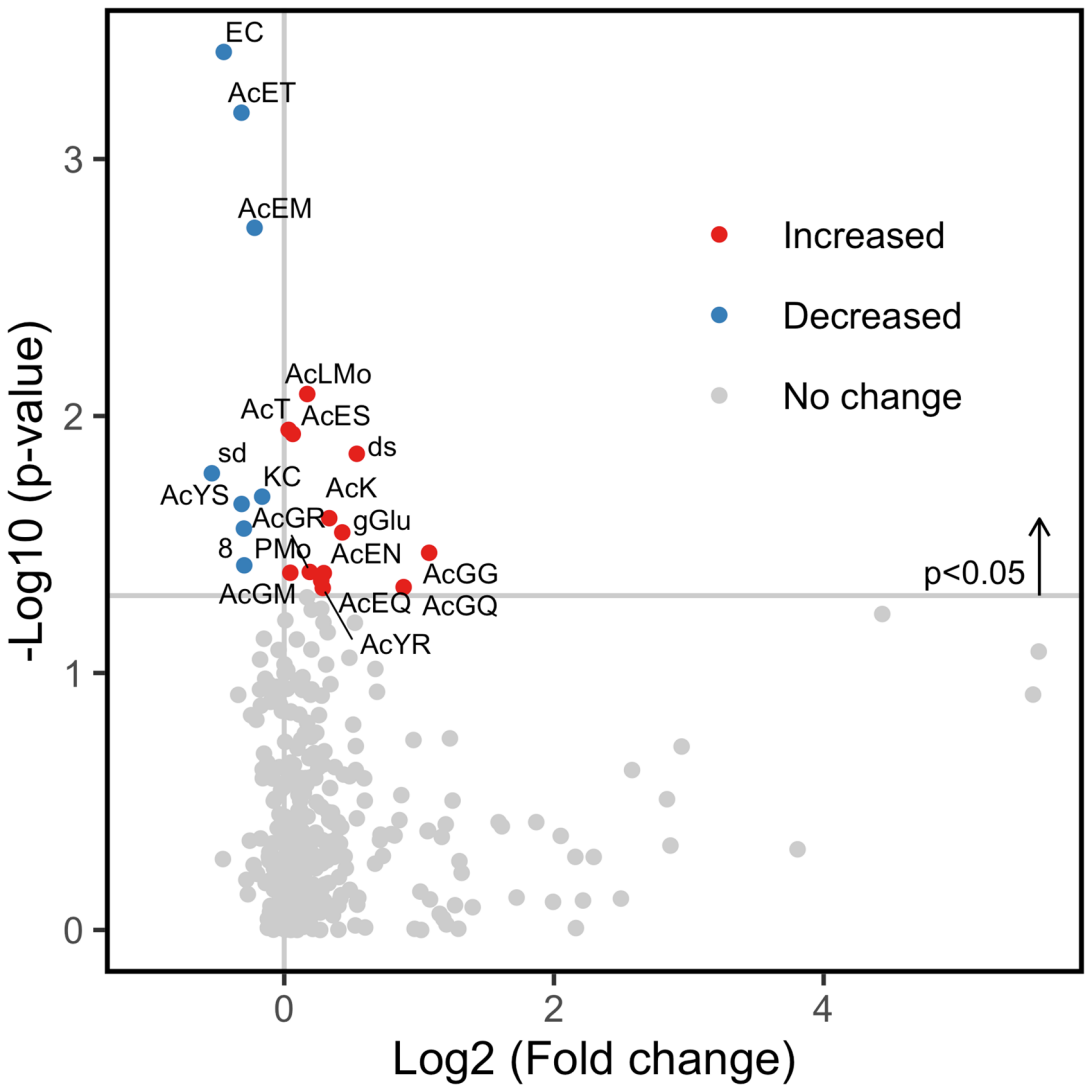
Fold change of enzyme activity in IBS-n patient fecal samples. Comparison of the enzymomic profiles between 35 healthy subjects and 35 IBS-n patients based on *t*-test. A total of 13 enzyme activities were increased (red circles) and 8 were decreased (blue circles) in IBS-n patients (raw *p* value <0.05) (shown above the horizontal line). Please refer to Supplementary Figure S1 for the abbreviations of the enzymes.

Next, the number of subjects was expanded to 70 (35 healthy subjects and 35 IBS-n patients), and the fluorescence intensity of 384 probes was measured 0.5 h after mixing the feces and probes. Multivariate analysis showed that some IBS-n patients had characteristic enzyme reaction patterns along the principal component 1 axis, but this was not the case for many other IBS-n patients, and no obvious clusters were apparent between healthy subjects and IBS-n patients (Figure 2; Supplementary Figure S3). Comparison of the fluorescence intensity of each probe between subjects showed that cleavage activity targeting 13 probes tended to increase and 8 probes tended to decrease in IBS-n patients (raw *p* value <0.05, Figure 3).

### 3.4. Fecal samples collected from IBS-n patients have increased trypsin-like activity and decreased elastase-like activity

**Figure 4:**
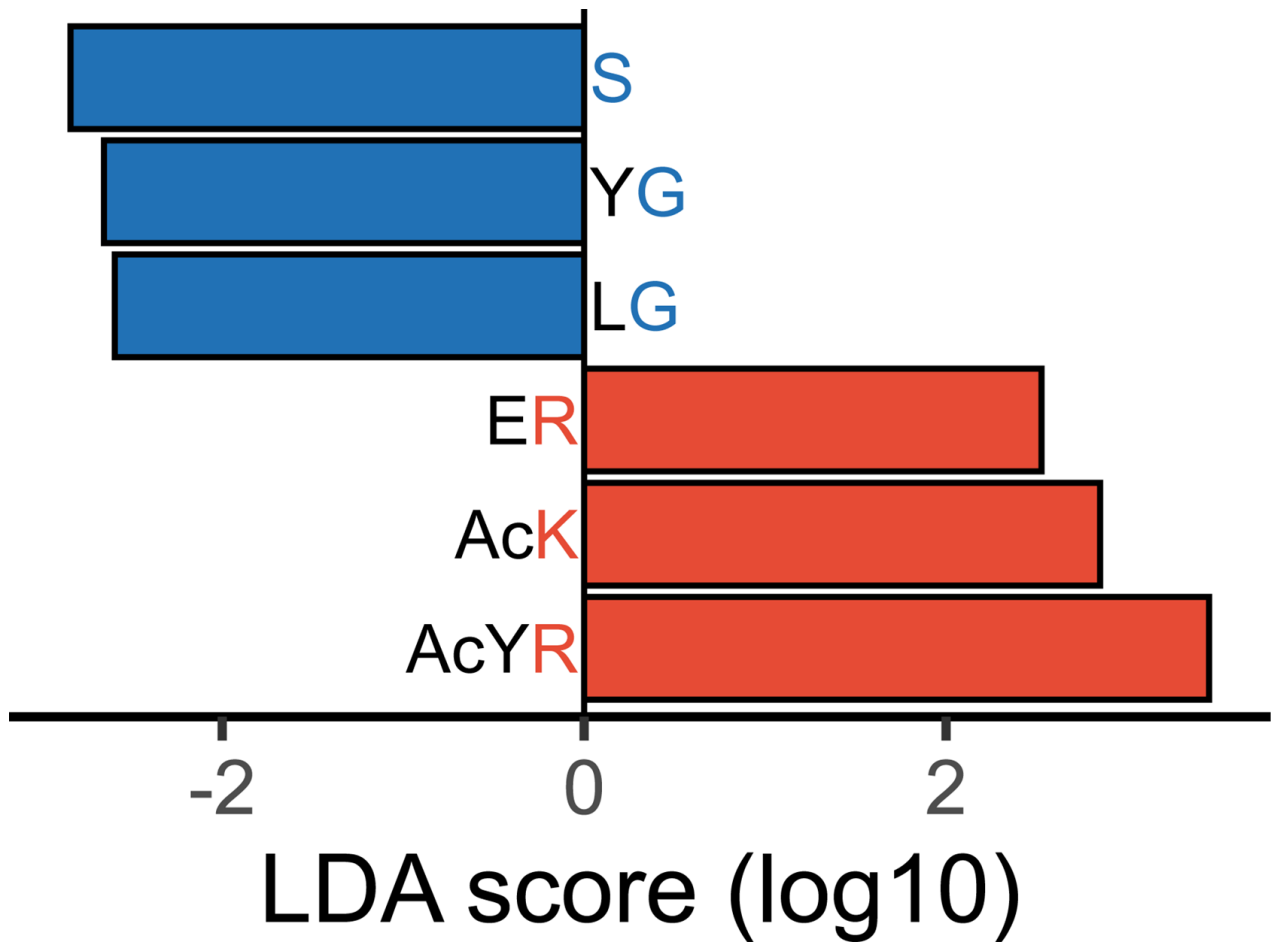
Differentiated enzyme activity in IBS-n fecal samples. Certain probes were differentially hydrolyzed in fecal samples of IBS-n patients. LEfSe analysis shows probes with | discriminant score | > 2.5, *p* < 0.05. Probes that were less actively cleaved in IBS-n patient fecal samples are shown in blue, and probes more actively cleaved in IBS-n patient fecal samples are shown in red. The alphabets represent amino acid residues. d = D-aspartyl, s = D-Serinyl, 8 = sarcosinyl, gGlu = γ-glutamic acid, Mo = methionyl-S-oxide, Ac = acetyl. For enzyme abbreviations, please refer to Supplementary Figure S1.

To distinguish between healthy subjects and IBS-n patients, linear discriminant analysis effect size (LEfSe) was performed using enzymomic data (Figure 4). LEfSe is an algorithm for high-dimensional biomarker discovery and explanation (Segata et al., 2011). The results showed that IBS-n patients had increased enzymatic activity for cleavage of peptide probes in which the C-terminal residue was a basic amino acid such as lysine (K) or arginine (R). However, the enzymatic activity for cleavage of probes terminating in relatively small amino acids such as serine (S) and glycine (G) was decreased in IBS-n patients. Notably, basic amino acids are cleaved by trypsin and small amino acids are cleaved by elastase, so we hypothesized that these observed differences in trypsin-like and elastase-like enzyme activity may be characteristic of IBS-n.

**Figure 5:**
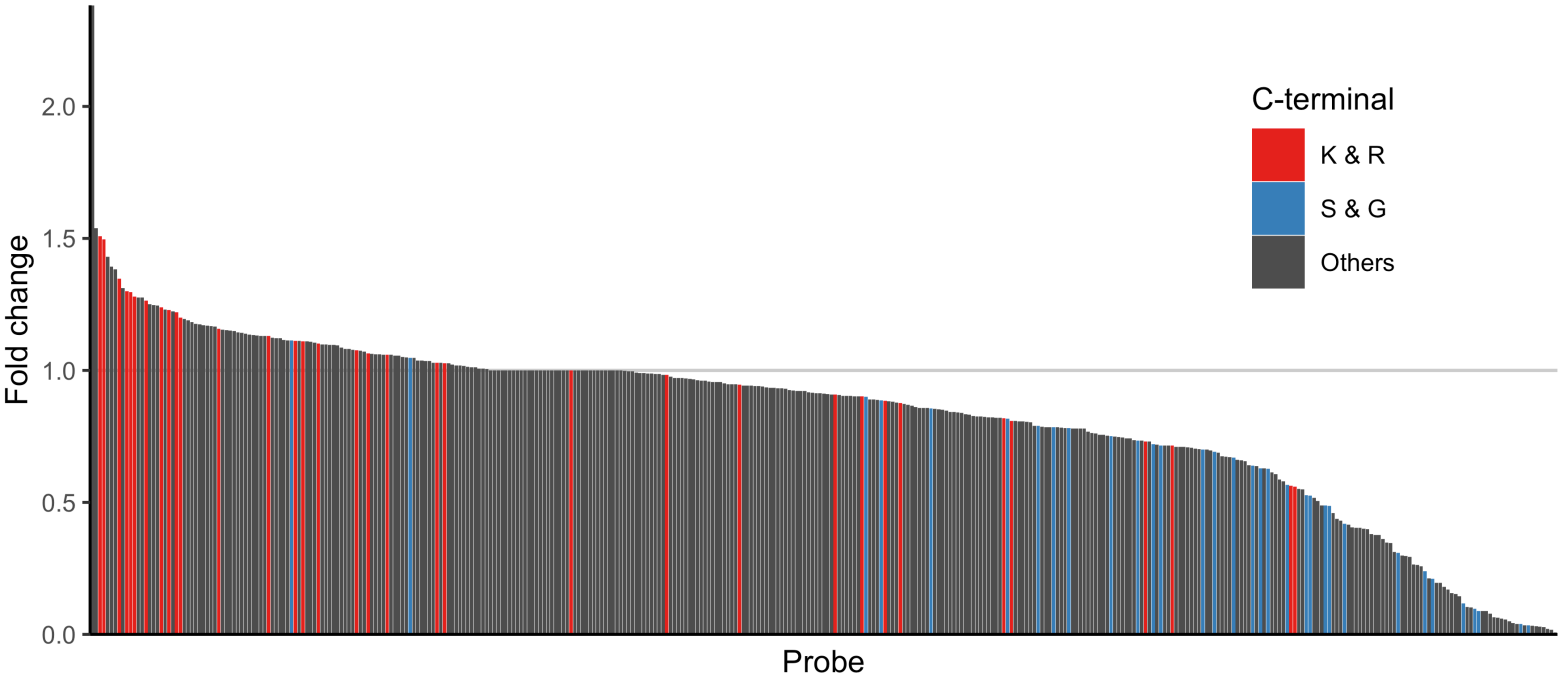
Comparison of enzyme activity between healthy and IBS-n fecal samples. The bar shows the fold change of normalized fluorescence intensity (IBS-n/healthy). These values were sorted by fold change, and the smallest normalized fluorescence intensity value except for 0 was added to all samples as pseudo intensity to calculate the fold change. The color shows the C-terminus of the probes: red for K and R, blue for S and G, and black for other probes. The alphabets represent amino acid residues. Ac = acetyl. For enzyme abbreviations, please refer to Supplementary Figure S1.

To determine whether probes with a C-terminal residue of K, R, S or G were cleaved differentially in fecal samples from IBS-n patients as the enzyme activity suggested, we examined the distribution of these probes among all probes. Consistent with the aforementioned results, the cleavage activity of the C-terminal K and R probes tended to be higher in IBS-n patients, whereas that of the C-terminal S and G probes tended to be lower in IBS-n patients (Figure 5).

### 3.5. Machine learning-based diagnostics using enzymomic profiles distinguish IBS-n patients with high accuracy

**Figure 6:**
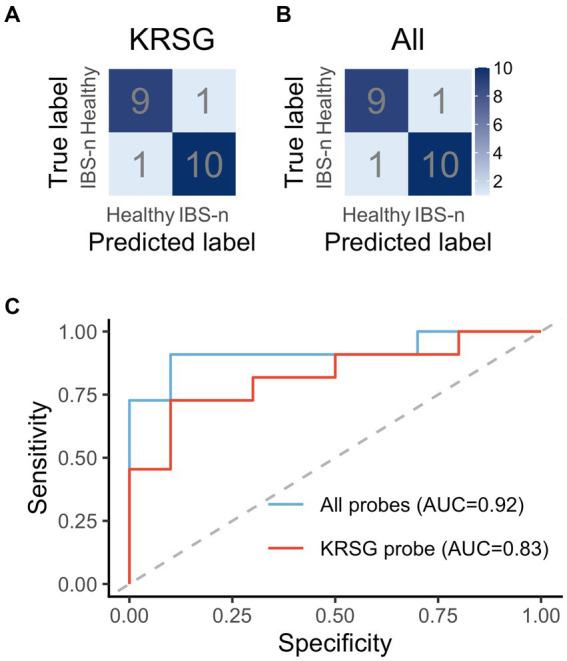
Performance evaluation of machine learning-based diagnostics. The confusion matrix shows the performance of the RF-based diagnostics using the probes with a C-terminal residue of K, R, S, or G **(A)** or all probes **(B)**. **(C)** ROC curves based on KRSG probes and all probes.

A machine-learning classification using RF was trained using selected probes that were shown to have significantly different reactivity between healthy subjects and IBS-n patients. Because RF decides the classification by majority vote in the forest (Uddin et al., 2019), there is a lower chance of overfitting the training data. Hence, this approach is useful when many predictors are noisy and can also be used when the number of variables is much larger than the number of observables (Manilich et al., 2011). The diagnostics trained using the data acquired with probes having a C-terminal residue of K, R, S, or G showed high accuracy (81.0%) and sensitivity (72.7%) (Figure 6A). Importantly, training with all probes resulted in better performance (90.5% accuracy, 90.9% sensitivity) (Figure 6B).

ROC analysis was performed to evaluate the robustness of the diagnostics (Figure 6C). The area under the ROC curve (AUC) of the diagnostics generated with the KRSG probes was high (AUC = 0.83). In contrast, the diagnostic performance of the metabolome data and gut microbiota data (Supplementary Figure S4) was low (AUC = 0.67 and 0.58, respectively). The robustness of the diagnostics generated with the data for all probes was also high (AUC = 0.92). Overall, these results showed that predictive performance was improved when all probes were used for analysis.

## 4. Discussion

To our knowledge, this is the first study to show that healthy subjects and IBS-n patients can be discriminated based on comprehensive fecal enzymomics profiling combined with a machine-learning approach. The most remarkable enzymatic reaction profile observed for IBS-n patients was an increase in trypsin-like peptidase activity to cleave C-terminal K and R residues and a decrease in elastase-like activity to cleave C-terminal S and G residues. While it has been reported that an increase in the production of trypsin-like proteases from the intestinal epithelium leads to increased trypsin activity in patients with IBS (Rolland-Fourcade et al., 2017), on the other hand, much remains unknown about the decrease in elastase activity. It is unclear whether this decrease is due to a reduction in elastase enzymes derived from the host or intestinal bacteria, or due to an increase in inhibitors. In recent years, there has been active performance of shotgun metagenomic analysis of the gut microbiota, and although understanding of the importance of the genes held by the gut microbiota has progressed, clarifying the protease activity as an actual function remains challenging. This methodology shows potential as a research tool for investigating the function of the host-gut microbiota. With these results as a starting point, it is likely that research will advance on understanding why elastase activity differs in IBS-n patients.

Conventional approaches have been able to identify specific enzyme effects (chymotrypsins, cathepsins, trypsins, and elastase-like activities) by quantifying mRNA and protein levels or measuring enzyme activity levels using probes, and it has been shown that trypsin-like enzyme activity increases in IBS-D patients (Cenac et al., 2007; Róka et al., 2007; Edogawa et al., 2020). However, even within trypsin-like activity, there are various types of trypsin-like enzymes. This study can identify not only enzyme activity in broad classifications, but also more detailed enzyme activity. Furthermore, this functionality-based analysis can reveal overall enzyme activity, including the presence of inhibitors, and it showed that net trypsin-like activity was indeed increasing in IBS-n patients. Like the other methods, this high-resolution and comprehensive enzyme activity measurement approach is applicable to other diseases besides IBS-n, and we believe that the approach can contribute to a better understanding of various diseases through data-driven research.

It is seen in the literature that a certain number of patients with IBS have low fecal elastase and are diagnosed with pancreatic exocrine insufficiency (Leeds et al., 2010; Talley et al., 2017). It has also been reported that pancreatic enzyme supplementation improves symptoms in IBS patients with low fecal elastase (Leeds et al., 2010). These findings are consistent with our results (Figure 5). On the other hand, they also reported that there was no significant association between fecal elastase and the diagnosis of IBS-D (Tooth et al., 2014; Talley et al., 2017; Arasaradnam et al., 2018; Black, 2021). This may be because IBS has a heterogenous etiology that has not been properly stratified. Also, our study measured activity, not amount of protease, so IBS patients may have normal amounts of elastase but increased amounts of endogenous elastase inhibitors (Vergnolle, 2016).

Which specific proteases increased the protease activity could not be identified due to the lack of well-characterized activity-based probes or fluorogenic substrates for detecting their activity and not just their expression. In this study, we also used various peptide probes whose C-terminal residues were basic amino acids to further elucidate the activity profile of the trypsin-like proteases involved. We found that most of the K and R probes showed increased activity in IBS-n patients, but several of the K and R probes showed decreased activity in IBS-n (Figure 5). Similarly, some of the S and G probes showed increased activity in IBS-n. Although we prepared probes with various C-terminal residues for efficient screening, future studies must utilize various non-C-terminal features to understand the role of these proteases.

Although we found that KRSG probe cleavage activity was altered in samples from IBS-n patients, diagnostic performance was improved by including other probes in the analysis (Figure 6). This indicates that machine learning can reflect various characteristics of heterogeneous physiological disorders, which is an advantage of a comprehensive analysis strategy. In addition, a principal component analysis showed a heterogenic population along the principal component 1 axis in IBS-n patients (Supplementary Figure S3). In fact, IBS-n patients can be stratified into clinical subtypes based on predominant symptoms (diarrhea, constipation, or mixed) (Jeffery et al., 2020). These results suggest the potential application of this strategy to identify IBS-D subtypes.

Our study had some limitations. First, the sample size was small. However, we could identify IBS-n patients with very high accuracy even with a small dataset of 70 subjects, demonstrating the practicality of our method. Second, our approach could only distinguish between IBS-n patients and healthy subjects. In clinical practice, however, it is especially important to distinguish IBS-n from inflammatory bowel disease, which has similar symptoms. Further studies dedicated to discriminating between IBS-n and inflammatory bowel disease and other diseases are needed to complement our findings and advance our understanding of the etiologies of these conditions.

In summary, comprehensive analysis of global enzyme activity in feces could accurately identify IBS-n patients in the absence of reliable biomarkers. This method enables non-invasive testing using fecal samples, a procedure traditionally employed in tests such as fecal occult blood tests. By utilizing a 384-well plate, this method provides higher throughput and is more cost-effective than endoscopic examination. We believe that this approach can be applied to other diseases for which biomarkers are not available, thereby lessening the burden on both patients and medical staff.

## Data availability statement

The raw data supporting the conclusions of this article will be made available by the authors, without undue reservation.

## Ethics statement

The studies involving human participants were reviewed and approved by the Tohoku University Hospital, Japan (2020-1-578). The patients/participants provided their written informed consent to participate in this study.

## Author contributions

SFa conceived the study and designed the experiments. KT conducted informatics analysis. NT performed enzynomics experiments. TK, YK, and YU provided fluorescent probes. YT and SFo collected fecal samples from IBS patients and healthy volunteers. KT, NT, IS, and SFa wrote the manuscript. All authors provided critical comments. All authors contributed to the article and approved the submitted version.

## Funding

This study was supported in part by JSPS Grant-in-Aid for JSPS Fellows (21J12844 to KT), JSPS KAKENHI (22H03541 to SFa), AMED-CREST (JP22gm1010009 to SFa), JST ERATO (JPMJER1902 to SFa), JST PRESTO (JPMJPR1537 to SFa), the Food Science Institute Foundation (to SFa), Taikichiro Mori Memorial Research Grants (to KT), the Ryoichi Sasakawa Young Leaders Fellowship Fund (to KT), and the Keio University Doctorate Student Grant-in-Aid Program from Ushioda Memorial Fund (to KT).

## Conflict of interest

SFa is a founder and CEO of Metagen, Inc., a company involved in the gut microbiome and metabolome-based human healthcare.

The remaining authors declare that the research was conducted in the absence of any commercial or financial relationships that could be construed as a potential conflict of interest.

## Publisher’s note

All claims expressed in this article are solely those of the authors and do not necessarily represent those of their affiliated organizations, or those of the publisher, the editors and the reviewers. Any product that may be evaluated in this article, or claim that may be made by its manufacturer, is not guaranteed or endorsed by the publisher.
