# Supplementary material for: A protease activity-based machine-learning approach as a complementary tool for conventional diagnosis of diarrhea-predominant irritable bowel syndrome
Source: Front Microbiol. 2023 Jul 7;14:1179534. doi: 10.3389/fmicb.2023.1179534 (PMC10361618; doi:10.3389/fmicb.2023.1179534)
Supplement: Supplementary file 1 [file Data_Sheet_1.pdf]

Supplementary Material

1 Supplementary Figures

A

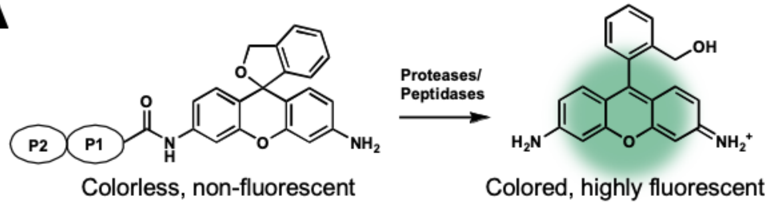

B

GG	EP	sP	ds	AcPG	AcPL	AcER	AcPH	EM	PQ	AcKT	D
EG	KP	bP	ss	E	P	AcKR	W	KM	AcQ	AcYT	GD
KG	YP	8P	bs	AcE	AcP	AcYR	GW	YM	AcGQ	AcLT	ED
YG	LP	Ga	8s	AcGE	AcGP	AcLR	EW	LM	AcEQ	AcPT	KD
LG	PP	Ea	Gb	AcEE	AcEP	AcPR	KW	PM	AcKQ	I	YD
PG	aG	Ka	Eb	AcKE	AcKP	A	YW	Mo	AcYQ	GI	LD
GE	dG	Ya	Kb	AcYE	AcLP	GA	LW	GMo	AcLQ	EI	PD
EE	sG	La	Yb	AcLE	AcYP	EA	PW	EMo	AcPQ	KI	AcD
KE	bG	Pa	Lb	AcPE	AcPP	KA	AcW	KMo	N	YI	AcGD
YE	8G	aa	Pb	K	F	YA	AcGW	YMo	GN	LI	AcED
LE	aE	da	ab	AcK	GF	LA	AcEW	LMo	EN	PI	AcKD
PE	dE	sa	db	AcGK	EF	PA	AcKW	PMo	KN	AcI	AcYD
GK	sE	ba	sb	AcEK	KF	AcA	AcYW	AcM	YN	AcGI	AcLD
EK	bE	8a	bb	AcKK	YF	AcGA	AcLW	AcGM	LN	AcEI	AcPD
KK	8E	Gd	8b	AcYK	LF	AcEA	AcPW	AcEM	PN	AcKI	C
YK	aK	Ed	G8	AcLK	PF	AcKA	S	AcKM	AcN	AcYI	GC
LK	dK	Kd	E8	AcPK	AcF	AcYA	GS	AcYM	AcGN	AcLI	EC
PK	sK	Yd	K8	Y	AcGF	AcLA	ES	AcLM	AcEN	AcPI	KC
GY	bK	Ld	Y8	AcY	AcEF	AcPA	KS	AcPM	AcKN	V	YC
EY	8K	Pd	L8	AcGY	AcKF	H	YS	AcMo	AcYN	GV	LC
KY	aY	ad	P8	AcEY	AcYF	GH	LS	AcGMo	AcLN	EV	PC
YY	dY	dd	a8	AcKY	AcLF	EH	PS	AcEMo	AcPN	KV	AcC
LY	sY	sd	d8	AcYY	AcPF	KH	AcS	AcKMo	T	YV	AcGC
PY	bY	bd	s8	AcLY	R	YH	AcGS	AcYMo	GT	LV	AcEC
GL	8Y	8d	b8	AcPY	GR	LH	AcES	AcLMo	ET	PV	AcKC
EL	aL	Gs	88	L	ER	PH	AcKS	AcPMo	KT	AcV	AcYC
8	dL	Es	AcG	AcL	KR	AcH	AcYS	Q	YT	AcGV	AcLC
KL	sL	Ks	AcGG	AcGL	YR	AcGH	AcLS	GQ	LT	AcEV	AcPC
YL	bL	Ys	AcEG	AcEL	LR	AcEH	AcPS	EQ	PT	AcKV	gGlu
LL	8L	Ls	AcKG	AcKL	PR	AcKH	G	KQ	AcT	AcYV	gPhe
PL	aP	Ps	AcYG	AcYL	AcR	AcYH	M	YQ	AcGT	AcLV	Boc-K(Ac)
GP	dP	as	AcLG	AcLL	AcGR	AcLH	GM	LQ	AcET	AcPV	HMRG only

a = D-alanyl, d = D-aspartyl, s = D-Serinyl, 8 = sarcosinyl, b = β-Alaninyl, gGlu = γ-glutamic acid, Mo = methionyl-S-oxide, Ac = acetyl, gPhe = glutamylphenylalanine,

Supplementary Figure S1. Construction of a fluorescent probe library. (A) Reaction scheme of dipeptidyl- hydroxymethyl rhodamine green with peptidases and proteases. P2 and P1 are the amino acids involved in the dipeptides that were tested. (B) List of the fluorescent probes in the library. The first and second letters represent the P2 and P1 positions of the amino acid residues. HMRG only is the control HMRG without an attached dipeptide.

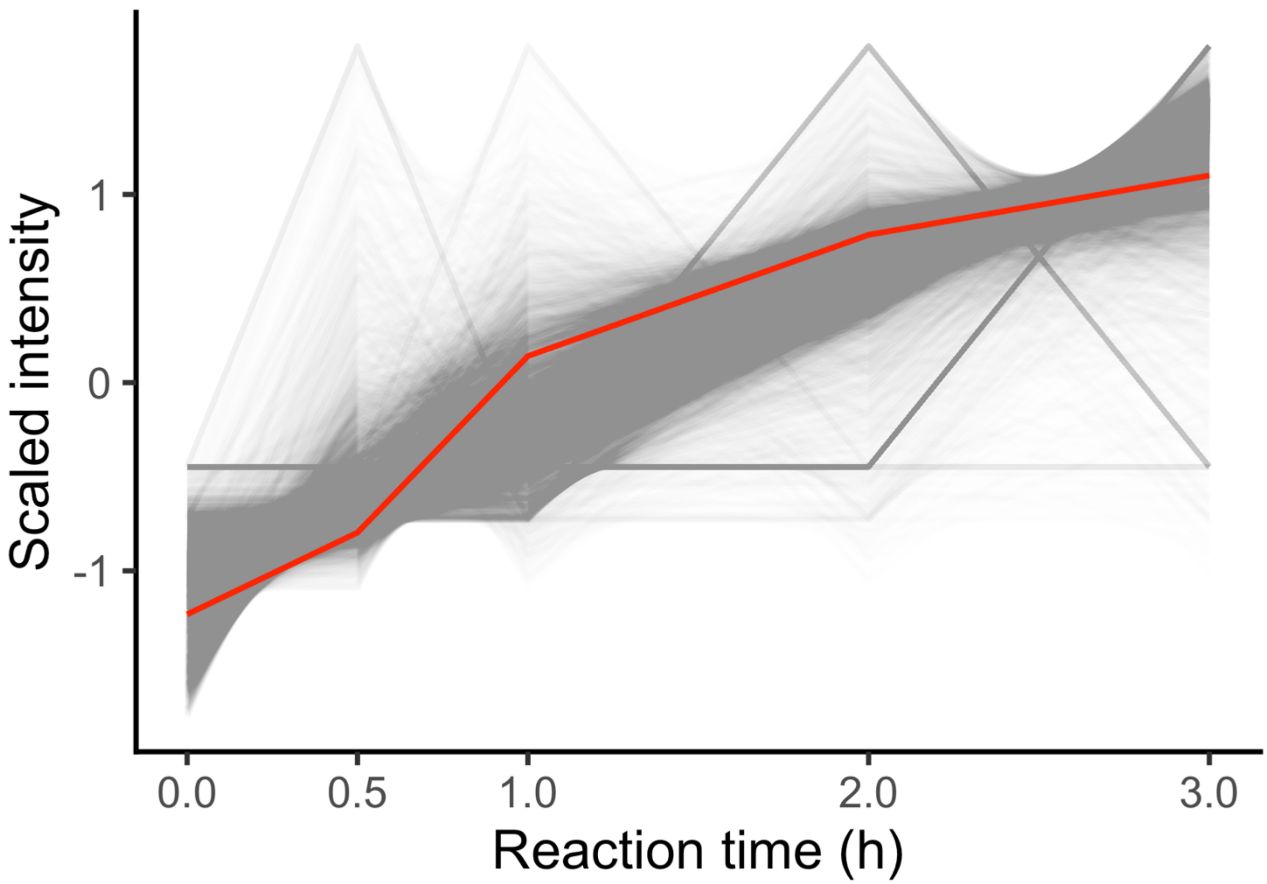

Supplementary Figure S2. Fluorescence intensity kinetics for determining minimum reaction time. To determine the appropriate reaction time, we analyzed the time-series change in normalized fluorescence intensity for a mixture of 20 fecal samples and 384 probes. The red line shows the median of all samples.

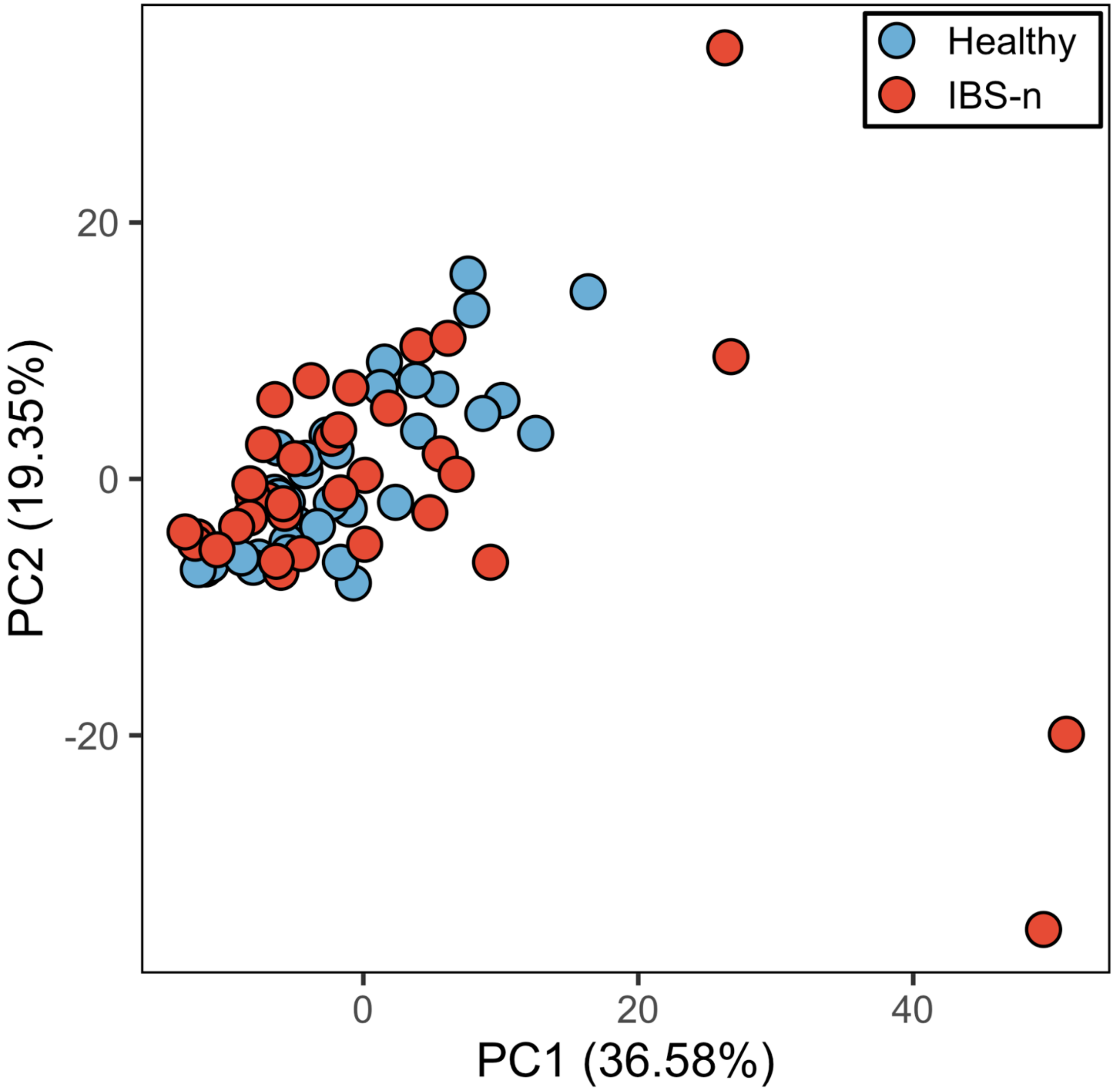

Supplementary Figure S3. PCA of probe fluorescence in healthy and IBS-n fecal samples. Analysis using fluorescence intensity data after 0.5 h of mixing 384 probes with feces samples collected from 35 healthy subjects and 35 IBS patients. The numbers in parentheses on the axes indicate the contribution ratios.

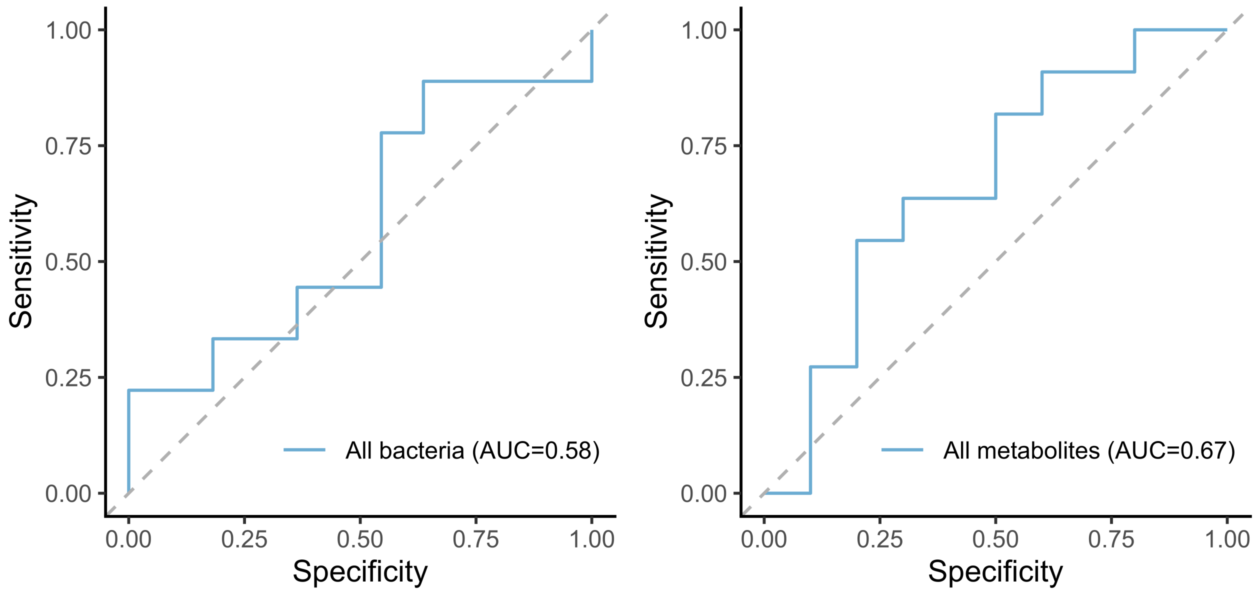

Supplementary Figure S4. Performance evaluation of RF-based diagnostics using metabolome and microbiome data. ROC curves based on metabolome and microbiome data.
